# Evolutionary force in confamiliar marine vertebrates of different temperature realms: adaptive trends in zoarcid fish transcriptomes

**DOI:** 10.1186/1471-2164-13-549

**Published:** 2012-10-11

**Authors:** Heidrun Sigrid Windisch, Magnus Lucassen, Stephan Frickenhaus

**Affiliations:** 1 Alfred Wegener Institute for Polar and Marine Research, Am Handelshafen 12, Bremerhaven, Germany

## Abstract

**Background:**

Studies of temperature-induced adaptation on the basis of genomic sequence data were mainly done in extremophiles. Although the general hypothesis of an increased molecular flexibility in the cold is widely accepted, the results of thermal adaptation are still difficult to detect at proteomic down to the genomic sequence level. Approaches towards a more detailed picture emerge with the advent of new sequencing technologies. Only small changes in primary protein structure have been shown to modify kinetic and thermal properties of enzymes, but likewise for interspecies comparisons a high genetic identity is still essential to specify common principles. The present study uses comprehensive transcriptomic sequence information to uncover general patterns of thermal adaptation on the RNA as well as protein primary structure.

**Results:**

By comparing orthologous sequences of two closely related zoarcid fish inhabiting different latitudinal zones (Antarctica: *Pachycara brachycephalum*, temperate zone: *Zoarces viviparus*) we were able to detect significant differences in the codon usage. In the cold-adapted species a lower GC content in the wobble position prevailed for preserved amino acids. We were able to estimate 40-60% coverage of the functions represented within the two compared zoarcid cDNA-libraries on the basis of a reference genome of the phylogenetically closely related fish *Gasterosteus aculeatus*. A distinct pattern of amino acid substitutions could be identified for the non-synonymous codon exchanges, with a remarkable surplus of serine and reduction of glutamic acid and asparagine for the Antarctic species.

**Conclusion:**

Based on the differences between orthologous sequences from confamiliar species, distinguished mainly by the temperature regimes of their habitats, we hypothesize that temperature leaves a signature on the composition of biological macromolecules (RNA, proteins) with implications for the transcription and translation level. As the observed pattern of amino acid substitutions only partly support the flexibility hypothesis further evolutionary forces may be effective at the global transcriptome level.

## Background

Marine ectotherms specialize to different thermal windows and undergo genomic changes under evolutionary forces seen as adaptation to environmental conditions. Temperature is a crucial abiotic factor causing seasonal variability in ecosystems and mainly distinguishing polar from temperate habitats, thereby determining the distribution of species on large scales [1].

Various molecular responses are described for seasonal temperature changes that range from alterations in the transcript amounts, enzyme activities and the resulting shifts in the energy- and housekeeping metabolism (for review see [2]). Furthermore, regulative mechanisms become effective in ectotherms, like the induction of specific heat shock proteins helping to adjust the metabolic functioning to seasonal warming [3]. In the cold, for example, glycoproteins are expressed to prevent freezing in subzero waters [4].

On longer time scales adaptation to temperature requires an effective housekeeping on the metabolic level (enzyme kinetics) as well as on the transcription level. An increase of protein numbers can only partly compensate for a loss in activity during seasonal acclimatization or permanent adaptation to cold, limited by constraints in cellular space and energy availability [5]. Hence, structural modifications at the protein level are necessary to improve enzyme operation in the cold. Different temperature adaptations and dependencies of the kinetics of single enzymes like the phosphoglycerate kinase [6,7], chitobiase [7] or the lactate dehydrogenase [8] were studied intensively complemented by comparative analyses at the amino acid sequence level [9], discussing thermal acclimation up to adaptation at the molecular level.

These studies described common principles of thermal adaptation in a limited number of enzymes and their three-dimensional structures. The flexibility hypothesis implies that thermal adaptation of enzymes is accompanied by shifts in catalytic turnover numbers (k_cat_) and catalytic efficiency (k_cat_/K_m_). For cold adaptation this demands a higher structural flexibility of the protein sometimes at the expense of reduced thermal stability [5,8,10,11]. For maintaining a certain degree of flexibility cold-adapted enzymes tend to have fewer salt links, less interactions within the hydrophobic core, a reduction in the number of proline and arginine residues, a reduction in the hydrophobicity of the enzyme, and improved solvent interactions with a hydrophilic surface via additional charged side chains. In most cases, the catalytic and binding centres are not changed, but mutations under adaptive pressure of temperature are changing the stability, the barriers of activation energy as well as the accessibility of the catalytic cleft through conformational changes in the structure [12].

Research on single enzymes demonstrated that several protein families adapt with different strategies, which even contradict with other observed patterns to some extent. After all, single case studies are not sufficient to identify global adaptive patterns in sequences at the DNA and protein level due to environmental temperature.

Recent genomic and proteomic studies on distantly related extremophiles display coherence between the optimal growth temperature (OGT) and the composition of biological macromolecules (DNA, RNA and proteins), in which higher GC contents prevail on a large scale in warm-adapted species [12-16]. Moreover, similar GC-trends were detected in eukaryotic synonymous sequences, when comparing poikilotherm and homeotherm species [15-17]. Subsequently a modified amino acid composition [13-15,17] of proteins can be observed, e.g., for non-synonymous mutations. In the present study we aimed to analyze evolutionary trends in marine ectotherm vertebrates of the same family based on transcriptomic data obtained from two normalized cDNA libraries. Since 454 sequencing-techniques are developed and improved, a number of transcriptomic libraries of various species were published, giving insights in the molecular networks comprising various developmental stages and tissue types [18,19]. The growing availability of sequence libraries allows for more specific inter-comparisons even in non-model organisms, e.g., by linking sequence data in closely related species in a pairwise manner.

The family of Zoarcidae (eelpouts) comprises 284 described species, distributed all over the global oceans [20]. Fishes of this cosmopolitan family are therefore appropriate to address the degree of adaptation to temperature in different realms by comparative methods. The eurythermal species *Zoarces viviparus* lives in the northern hemisphere and faces seasonal shifts of temperature ranging from 0°C in winter to above 20°C in summer [21]. Despite, the maximal growth rate for this species was determined at about 15°C, indicating an optimal ecological and physiological performance at this temperature [22]. Kristiansson and colleagues sequenced a transcriptomic cDNA library of *Z. viviparus* under unstressed conditions [23]. Furthermore, this species is often compared in ecophysiological studies with its stenothermal Antarctic congener *Pachycara brachycephalum*[24] that is highly adapted to constant cold waters around 0°C and an optimal growth temperature at 4°C under laboratory conditions [25].

We constructed a transcriptomic cDNA library from liver and heart muscle tissue of the Antarctic eelpout *P. brachycephalum* under habitat conditions and compared it with a cDNA-library of *Z. viviparus* at functional level, their amino acid sequence translations and at RNA codon level to uncover differentiation, which we can explain at best by thermal adaptation.

## Results

**Figure 1:**
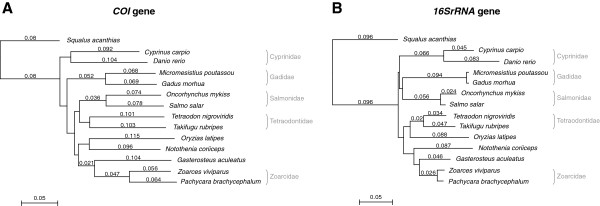
**Phylogenetic trees of representative fish species inhabiting divergent temperature realms. A**: Sequence similarity based on the *COI* barcoding gene. **B**: Phylogenetic map based on the sequence information of the structural *16SrRNA* sequence. Both trees were generated by using the neighbour joining method. Node distances are calculated with Tamura-Nei algorithm. Numbers indicate relative nucleotide substitutions in which different substitution pathways occur with independent probabilities. Sequence gaps were distributed proportional. A summary of the used sequence information is summarized in material and methods Table 3.

A classification of *P. brachycephalum* and *Z. viviparus* by molecular analyses of selected sequence data was used to determine the phylogenetic relationship with various bony fish species from widespread geographical regions. For two mitochondrial genes (*ribosomal 16S RNA* (*16SrRNA*) and *cytochrome-c-oxidase subunit I* (*COI*)) we computed phylogenetic trees of 13 selected species belonging to the class of Actinopterygii and the elasmobranch *Squalus acanthias* as outgroup. Both zoarcids formed a clade (Figure 1A, B). The threespined stickleback *Gasterosteus aculeatus* appeared as their closest relative for both genes. The obtained phylogenetic trees mirror the grouping into the different families like Cyprinidae, Gadidae, Salmonidae, Tetraodontidae and other Percomorpha. Quantitatively, higher similarities across various species were found for the *16SrRNA*, i.e., smaller distances, than for the *COI* gene. From both trees a close relationship could be inferred among the zoarcid species, although *COI* is a functional gene and the *16SrRNA* is a structural RNA. We expect similar differences to be present also in sequences of functional genes. In this context a manual pre-selection of a limited subset of sequences would bias a comparative study. To this end a more complete approach is followed here based on all available transcriptomic sequences of the closely-related zoarcid fish. 

### Overall statistical comparison of zoarcid cDNA libraries

**Figure 2:**
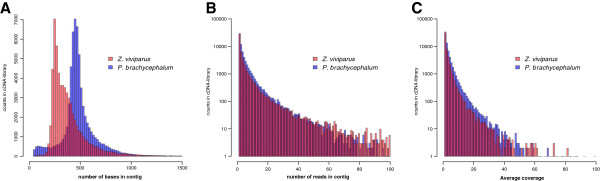
**Statistical analyses of the sequencing runs and the associated assembly quality. A**: Distribution of the contig length of *P. brachycephalum* and *Z. viviparus.***B**: Number of reads in the assembled contigs. **C**: Average coverage of reads in assembled contigs.

The setup comprises 454 sequencing data of two cDNA libraries, one prepared from liver and heart of *P. brachycephalum,* and one prepared from liver of *Z. viviparus*[23], generated with similar techniques and protocols (c.f. Material and Methods). The average read length in the library of *P. brachycephalum* was about 100 bases longer than that for *Z. viviparus* (Table 1), due to the usage of Titanium-chemistry for sequencing. Subsequently, a higher yield of longer contigs with 487 bases in average for *P. brachycephalum* was found, compared to 342 bases in average per contig for *Z. viviparus* (Figure 2A). Other basic parameters like number of reads per contig (Figure 2B) as well as the average coverage were comparable (Figure 2C), constituting a suitable basis for alignment analyses of contigs. 

**Table 1:** Benchmark data of cDNA libraries

**Database parameter**	***P. brachycephalum***	***Z. viviparus***
Total Number of reads	481,802	~400,000
Number assembled	338,993	349,102
Average of read length	321	221
Number of all contigs	65,565	53,447
Number of non-redundant contigs	64,634	53,313
Percentage of redundancy	1.42	0.25

Overall statistical analyses of the sequencing runs of *Z. viviparus* data of the library generated by Kristiansson et al. 2009, in comparison to the library of the Antarctic eelpout *P. brachycephalum*. The resulting reads were set up to contigs with similar assembly techniques. Further analyses of the contig quality could be seen in Figure 2.

**Figure 3:**
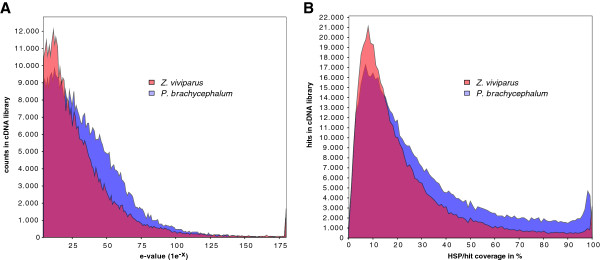
**Overview of the distribution of BLAST results among the library assemblies with BLAST2GO. A**: the e-value distribution shows the hit-reliability of the contigs **B**: The HSP (high-scoring segment pair) distribution gives a measure of the accuracy of the sequence alignment within the BLAST annotation.

Using the BLAST2GO tool [26] for evaluating annotations of contigs, statistics of expect values (e-values) show qualitatively comparable profiles, But quantitatively, sequences of *P. brachycephalum* show higher similarity to known sequences (Figure 3A) accompanied by higher coverage per hit of high-scoring segment pairs (HSP) (Figure 3B). For the purposes of this study we filtered by BLAST [27] for sequences of known functions, i.e., showing known translations, resulting in 19,460 contigs for *P. brachycephalum* and 16,315 for *Z. viviparus*. 

### Functional coverage compared to a phylogenetically related reference genome

**Figure 4:**
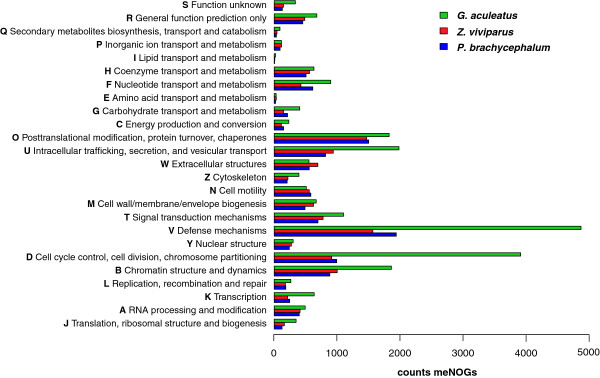
**Distribution of metazoan orthologous terms in clusters of orthologous groups (COG) determined by rpstBLASTn (e-value cut off 10**^**-20**^**) in the transcriptomic libraries of *Z. viviparus *and *P. brachycephalum. ***Complete genomic data of *G. aculeatus* representing a reference from a closely related fish were analyzed equally.

The overall functional repertoires of the cDNA library sets were evaluated in terms of KOG/COG categories (clusters of orthologous groups) as obtained from the local alignment tool rpstBLASTn applied on the BLAST-annotated sequences against a database of metazoan-specific orthologies (meNOGs) [28,29]. As a scale for the comparison, coding sequences from the closely related species *G. aculeatus* (Figure 1A, B) were evaluated accordingly. From the reference genomic data (ENSEMBLE 61) 27,628 coding sequences were extracted to estimate the functional coverage of zoarcid libraries. To compare the appearance of functional terms quantitatively, the counts were pooled into superordinated categories of KOG/COGs (Figure 4). The count data of both zoarcid transcriptomes were in a comparable range but covered only a fraction of the reference genome. Strongest inter-zoarcid differences were seen for categories F (nucleotide transport and metabolism) and V (defense mechanisms), showing higher counts in *P. brachycephalum*. The overall coverage compared to the scales of *G. aculeatus* genome is estimated to be 40% to 60%, with a pronounced lesser coverage in categories U, V, D and B. Furthermore, a notable deviation is found in category N (cell motility), where both eelpouts show more counts than are present in the reference genome. Contrary, in category W (extracellular structures) *Z. viviparus* have more counts compared to the reference set, whereas the number of counts for *P. brachycephalum* nearly equals it. 

To identify species-specific functional patterns in gene expression, we searched for overrepresented meNOGs in each library set. The most exclusive terms in *Z. viviparus* are meNOG13752 coding for complement protein, meNOG11371 encoding for the cytochrome-*c*-oxidase (COI) and meNOG18150 for extracellular matrix constituent lubricant protein contributing to the structural extracellular matrix integrity. In *P. brachycephalum* the most specific term is a b-box-type zinc finger (meNOG24554) followed by meNOG15201 encoding for a tripartite motif protein and meNOG06310 which encodes a carboxylase function. Further terms in this species were ubiquitin (meNOG05057) and the heavy chain of myosin (meNOG 07153) (data not shown). The term for myosin (meNOG07153) is noticed as specific for *P. brachycephalum* consistent with the fact that we included heart RNA in the library construction.

### Amino acid usage

**Figure 5:**
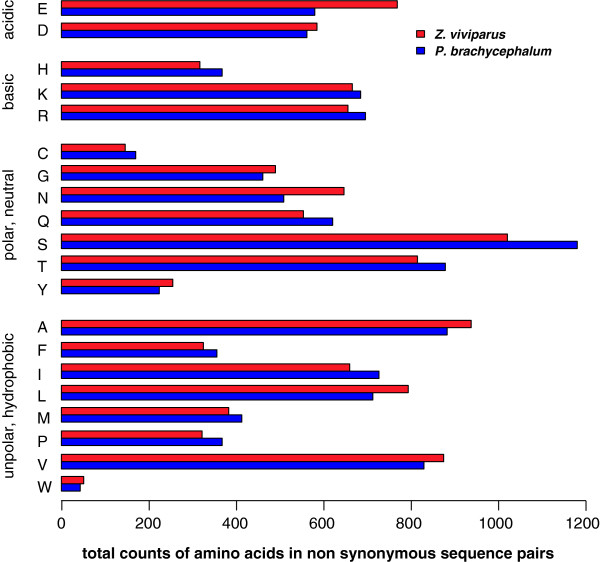
**Frequency of differential amino acid usage based on the sequence alignments of the cDNA libraries with fish-specific orthologies. **Local alignments of resulting 4,155 sequence segment pairs were analyzed for net amino acid usage in non-synonymous translations.

Translations of coding sequence segments were generated based on fish-specific orthologies (fiNOGs) via rpstBLASTn for both cDNA libraries to exclude effects of sequencing artefacts and frame shifts on the subsequent analyses. From the identified orthologous sequences in each zoarcid fish, matching sequence segment pairs were obtained by harvesting best BLASTp hits per model orthology (i.e. fiNOG), providing a set of shared orthologous sequences in both fish. For a more stringent pairing, i.e., excluding probably false pairs, translated segment pairs were filtered for ≥ 80% amino acid sequence identity. Accordingly, frequencies of amino acids were computed from 4,155 translated and realigned segment pairs, including non-identical amino acid positions, but excluding indels (insertions/deletions). Alterations in amino acid profiles became visible by assessing the total changes in the aligned non-synonymous codon positions (Figure 5). For *P. brachycephalum* net losses for glutamic acid and asparagine as well as a net gain of serine were most obviously. 

To resolve the exchange patterns in more detail, a table of amino acid replacement counts was transformed to a measure of imbalance of amino acid replacements in *P. brachycephalum* compared to *Z. viviparus* (see Methods and Table 2). Similarly, the most striking difference is the loss of 85 glutamic acid in *P. brachycephalum* with a preferential usage of aspartic acid instead. Further patterns of prominent exchanges are the replacement of 31 leucine by alanine, likewise on a similar level the exchange of 30 glutamic acid residues by glutamine. Serine is used preferentially for almost all amino acids but only replaced by arginine (30 cases). The observed patterns for threonine are not only the preferential usage instead of 82 alanine and 41 asparagine but also replacements for 46 methionine and 31 serine. Beside an overall net loss, valine is preferred over alanine 51 times but also replaced by 65 isoleucine. 

**Table 2:** Imbalances of amino acid usage in two zoarcid species

		**Amino acids preferred in *Z. viviparus***																		
		**A**	**C**	**D**	**E**	**F**	**G**	**H**	**I**	**K**	**L**	**M**	**N**	**P**	**Q**	**R**	**S**	**T**	**V**	**W**
**Amino acids preferred in *P. brachycephalum***	**C**	3																		
	**D**	−10	−1																	
	**E**	−19	−1	−85*																
	**F**	−3	−3	1	0															
	**G**	−13	−8	7	5	−1														
	**H**	0	−1	9	−3	−2	5													
	**I**	−5	−1	2	0	−15	0	−6												
	**K**	−11	1	7	15	−2	−2	−4	2											
	**L**	−31*	−3	3	−3	26	−4	−9	−25	2										
	**M**	5	−4	3	−2	−10	−1	−3	−20	−5	19									
	**N**	−18	1	25	−1	−8	−16	−21	−7	−23	2	2								
	**P**	3	0	1	2	6	4	0	−1	−3	−6	−5	5							
	**Q**	7	2	5	30*	0	20	7	1	−11	9	−4	18	−5						
	**R**	5	−8	0	18	−1	8	−10	0	−7	−5	−3	0	−25	22					
	**S**	5	11	2	26	19	8	1	9	20	11	2	15	3	6	−30*				
	**T**	82*	12	21	−5	−2	−1	0	7	6	3	−46*	41*	−7	−5	−4	−31*			
	**V**	51*	−8	3	3	−19	1	−1	−65*	7	−11	7	1	−6	−3	−7	−10	11		
	**W**	1	0	0	0	−4	−5	0	0	0	12	−1	0	0	−5	−3	−7	0	3	
	**Y**	3	−10	8	−1	−23	2	3	7	1	3	0	−6	0	−3	−2	−4	−4	−4	−1

In 4,155 orthologous sequences amino acid usage was analyzed by aligning the fiNOG-orthologous sequences of *P. brachycephalum* and *Z. viviparus.* The algebraic sign shows the direction of the imbalance of differential usage. Negative signs show a loss, positive signs show a gain of the corresponding amino acid in the Antarctic species. Most evident changes were emphasized and labelled with an asterisk.

The observed imbalances were furthermore reanalyzed taking the ERK–proxy into consideration [30] to determine whether species-specific shifts are detectable within the observed pattern of differentially used amino acids. A significant preference for using E+R+K in *Z. viviparus* was detected when compared to the orthologous associate in *P. brachycephalum* (Wilcoxon test, p = 0.02), analysing the 4,155 translated sequences and taking only exchanged positions into account.

### Codon usage

**Figure 6:**
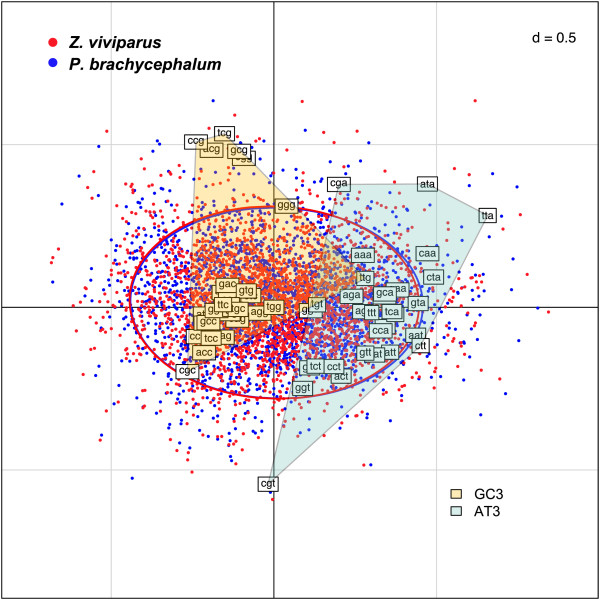
**Within Canonical Analyses. **All codons of the synonymous sequences of *P. brachycephalum* and *Z. viviparus* were plotted in a factorial map depicting the different counts for all combinations. The left polygon is comprising the codons with a G or C on the third position, the right polygon envelops those ending with A or T. Ellipses were computed from covariance estimates, characterizing the average canonical positions of the two sequence sets.

**Figure 7:**
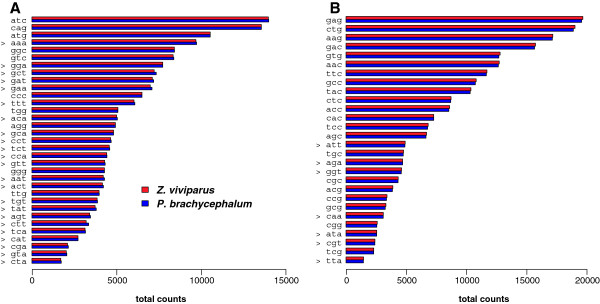
**Differential usage of amino acids in the zoarcid species. **4,155 homologue sequence pairs were used for the analyses. All codons with A/T in the third position are labelled with ‘>’; **A**: The panel displays codons with higher usage frequencies in the Antarctic species; **B**: Complementary plot for higher usage frequencies in the temperate species.

The set of 4,155 accordingly re-aligned orthologous coding sequence pairs in the transcriptomic eelpout-libraries were analyzed by means of a Within Canonical Analysis (WCA) to distinguish synonymous codon usage between the two species [31]. Comparing the composition of the triplets coding for synonymous amino acids, a shift of the *P. brachycephalum* transcriptome sequence segments became visible in the direction of a preferred usage of A or T in the third position of codons (Figure 6). The small shift between the blue covariance ellipse relative to the red one is exclusively due to the first principal component, reflecting the G/C-content on the third codon position (GC3). Furthermore, with codon usage differences from 4,155 sequence segment pairs (filtered for > 80% identical translated positions, depicted in Figure 7), more positive shifts (23 in the left panel) of codons ending with A/T (AT3, marked “>”) were detected compared to the amount of negative shifts (Figure 7 in the right panel). For this analysis, non-synonymous codon positions were not taken into account. A Fisher-test for the distribution of a contingency table of AT3 vs. GC3 codons in the left and right panel of this graph reveals a p-value of 5*10^-4^, indicating a significantly non-random distribution of shifts. 

The overall number of aligned amino acid residue pairs in this set was 414,978, constituting an average of 100 aligned codon pairs per segment with a mean of 96 conserved residues. Comparing 402,219 synonymous codon pairs with conserved nucleotides in positions 1 and 2, *P. brachycephalum* shows a net GC3-loss of 1,030, re-confirming an imbalance towards a preferential A/T in the third position of the codons in this cold-adapted fish*.* The analysed coding sequence segments showed a mean GC-fraction of 53.196% for *Z. viviparus* and 53.094% for *P. brachycephalum* mirroring the observed pattern on the third codon position.

## Discussion

In the present study we profiled transcriptomic libraries of two confamiliar fish species from different temperature realms for signatures of thermal adaptation at the sequence level. The phylogenetic analyses (Figure 1A, B) indicate a close relationship between *P. brachycephalum* and *Z. viviparus*, which is supported by transcriptomic sequence comparisons finding a pool of 4,155 comparable sequence segments with a mean identity of 96%. As only minor sequence changes are sufficient to adapt kinetic properties of orthologous proteins, this tight relationship is essential for identifying a potential thermal signature at the genomic and transcriptomic level in species from thermally distinct habitats.

Habitat temperature may be an important trigger for the observed patterns. As *Z. viviparus* faces similar cold temperatures in winter like *P. brachycephalum*, a signature for thermal shifts may appear. As optimal growth performance constitutes an important marker for maximal protein synthesis, which was determined for the upper third of the temperature window [22,25], we postulate that the transcriptome is optimized to this temperature range and not to the cold edge, where metabolic activity and performance of the eurythermal species is comparably low.

It is important to note the assumptions we made to argue that the observed changes are adaptive. Garland and Adolph [32] generally argued against comparisons of only two species for making appropriate conclusions about adaptation, since the probability for finding a difference for any trait between two species is always 50% and no valid null hypothesis can be made. According to their critical statement [32], the limitation could be overcome by analysing several traits (about 5 independent) as this will reduce the type I error rate to the excepted rate (p < 0.05). As we used several thousand mRNA/protein sequences for the analyses from the beginning, we can assume that there are still enough independent traits left to reach a p < 0.05. This is essentially true for the analyses of the synonymous codon exchanges, which have no impact on function and can be stated as “neutral” at best. For the non-synonymous codon exchanges we can postulate the same, as the exchange of one specific amino acid in one protein will not cause a definite amino acid in another protein, even if both proteins belong to the same trait. If only a genetic drift has taken place after separation of the two species from the common ancestor, no change in codon or amino acid usage would be expected when analyzing thousands of traits. Since we found a clear signal, we have to conclude that this change is adaptive. The question remaining to be answered is whether the driving force is temperature (alone) or a combination with further factors (see below).

Codon and amino acid usage were found highly correlated with expression levels at least in fast growing bacteria and yeast [33]. Specific needs of an organism may shape a specific codon-usage-profile with a concomitant optimization in the translation apparatus (tRNA amount, stability of secondary structures of RNA, amino acid levels, etc.). Here, we compared qualitatively the profiles of two (related) species. The normalized cDNA libraries were synthesized with the same protocol by the same laboratory but sequenced with different procedures. Nevertheless, both libraries proved to be comparable on the level of transcript diversity (Figure 2) as well as qualitatively on the level of attributable functions (c.f. Figure 3). Comparable subsets of transcriptomic sequences comprising reliable sequence information for alignments on amino acid and RNA level were generated based on the BLAST results, covering 40 to 60% of functions seen in a related reference genome. In summary, shifts in codon usage between the species seem not biased by expression level per se but possibly by evolutionary forces requiring specific expression levels in a certain (thermal) environment. This aspect is not further addressed within this study.

### Functional coverage compared to a reference genome

It is noteworthy that translated coding sequences of *G. aculeatus* are part of the eggNOG orthologies. Therefore, we expect that a comparison in a subset of these orthologies, i.e., metazoan (meNOGs), is useful to serve as a scale for inter-comparisons of the present cDNA libraries. Nevertheless, comparisons with *G. aculeatus* genomic coding sequences might show an overestimation of coverage due to the unresolved multiplicities by splice variants present in the assemblies of the transcriptomes. This fact might explain the observed higher coverage of functional terms in the KOG/COG categories N (cell motility) and W (extracellular structures) of the zoarcid fish compared to the reference genome.

Screening for occurrence of library-specific terms in principle allows for detection of signatures of the environmental conditions under which the transcriptomes were obtained, and not necessarily indicates presence or absence of genes in one of the species genomes. As a result transcripts related to *COI* were detected in elevated proportion in *Z. viviparus*, which is against the expectation that the normalization step in library preparation should reduce over-abundant RNA-variants in both libraries to a comparable degree. The same holds for the Antarctic species, for which the frequency of ubiquitin annotation appears in a higher rank. Discovering library specific functional terms, which would be expected to be included in similar ranks in both transcriptomes, reflects differences in the efficiency of normalization.

Other terms, specific for the cDNA library of *Z. viviparus* are related to complement protein (meNOG13752) and extracellular matrix constituent protein (meNOG18150). These terms summarize functions for immune response and integrity of the tissue, comprising cell communication, growth factors and wound healing. The term for tripartite motif (TRIM) proteins, which is predominantly found in the *P. brachycephalum* library, summarizes sequences of a protein family associated to pathogen-recognition, regulation of the concomitant transcriptional responses, constituting an important part of the immune system [34]. These differences may indicate adaptive forces acting on the immune system or simply different life histories of the sampled specimens. Responses of the innate immune system in fish were apparently more robust and diverse than in higher vertebrates [35]. Similarly, e.g., Atlantic cod comprises a unique immune system with substantial gene losses in adaptive components without being exceptionally susceptible to disease under natural conditions [36]. Consequently specific adaptations in the immune systems in the two eelpout species due to the different habitat conditions seem possible.

Another overrepresented term in *P. brachycephalum* is a carboxylase, which belongs to the KOG/COG category I - lipid transport and metabolism, as listed in the meNOG tables. The lipid fraction in liver of *P. brachycephalum* kept at 0°C is threefold higher than in *Z. viviparus* at habitat temperature [37], in line with the general finding that Antarctic species have high capacities for catabolism of fatty acids [38]. Furthermore, a recent study demonstrated relationships between preferred energy fuels and transcript levels of respective genes in *P. brachycephalum*[39]. Although the overrepresentation of the carboxylase fits into the current picture, further genes could have been expected to occur substantiating this evidence. This mismatch may diminish when the entire genome sequence data will become available. In summary, some of the library specific functions identified in our study may point to transcriptomic and possibly genomic contrasts due to the different environmental conditions of the discrete habitats of the two zoarcid fish under study.

### Amino acid usage

With the advent of more genomic data from species from various habitats analyses of temperature effects on amino acid usage profiles and the correlated GC content became possible [13,40]. Gu and Hilser [41] analyzed intra-protein interaction energies on the level of primary and secondary structural sequence segments in thermophilic and psychrophilic proteomes, supporting the flexibility hypothesis of cold adaptation. Furthermore, local and global adaptation patterns were differentiated, depending on different protein families.

By comparing the amino acid composition of a comprehensive set of orthologous sequence pairs globally we discovered a distinct pattern of replacements, which may result in structurally important changes in the functional structure of proteins supporting cold adaptation of primary protein structures (Figure 5, Table 2). In the following section all observed changes of amino acid usage are discussed in respect of the Antarctic species when compared to the *Z. viviparus* transcriptome.

The most obvious shifts found for *P. brachycephalum* are the net loss of glutamic acid and asparagine and the gain of serine (compare values of Table 2 and Figure 5). The frequency of all acidic residues is reduced and contrasts with slightly higher frequencies for the basic amino acids lysine and arginine as well as histidine. Due to the eminent loss of glutamic acid, the total amount of charged residues for stabilizing salt bridges is reduced. This may contribute to weaker interaction involved in substrate binding and protein interaction. The net loss of glutamic acid and a preferential exchange with 85 aspartic acid residues preserves the acidic function at reduced flexibility of the side chain. These exchanges may cause increments in charge density at the surface favouring increased solvent interaction as postulated earlier [11,42]. A reduction of salt links results from the exchange of the charged glutamic acid by polar glutamine (30 Q over E), in line with earlier assumptions drawn from analyses of individual proteins [11,42]. Arginine as a bulky amino acid was identified to be less abundant in cold-adapted species [11,42]. However, replacements of 30 serine into arginine and a slight increase of arginine on a global level contrasts with both former assumptions.

Based on genomic comparisons Wang and Lercher [30] developed a simple, reduced predictor based on replacement frequencies of the charged amino acids glutamine (E), arginine (R) and lysine (K), which were found most often exchanged in a thermal cline. Higher values for the ERK-proxy are found to be characteristic for hyperthermophiles compared to thermophiles and mesophiles. However, the ERK signal in our study is purely based on the large E change (Figure 5), as a minor change of R+K in the reverse direction does not confirm the ERK-hypothesis perfectly. We interpret this partial contrasting outcome as a consequence of extrapolation, i.e., application of a thermophile-mesophile hypothesis below the mesophile scope seems to be appropriate only for the E signal. Furthermore, we are aware of the finding of Lobry and Necsulea [40] that thermophile-mesophile signatures of cold adaptation are more prominent [43] than mesophile-psychrophile signatures, at least in analyses of codon-usage trends in coding sequences of complete genomes.

Within the group of polar amino acids the picture is heterogeneous due to the large gain of serine and the net loss of asparagine. Comparing the total counts of gains and losses of polar with unpolar residues a gain of 102 polar residues remains. This indicates that despite this pooling unpolar to polar shift -supporting increased solvent interaction- cannot be resolved as a strong signal within our study.

By comparing a limited set of prokaryotic proteome sequences amino acid frequencies canonically discriminating psychropilic, mesophilic and thermophilic species were determined after modelling of candidate genes on existing three-dimensional structures [43]. In this way the analysis of particular orthologuous sequences were extended to part of the proteomes, and amino acid frequencies were assessed separately in structural categories ‘buried’ and ‘surface’. In general, a trend towards polar residues (in particular serine) resemble the finding of a (solely significant) preferential usage of serine and a net gain of polar residues for a cold-adapted species. Our study confirms these findings as a cold induced positive serine shift is one of the dominating signals in our study. Therefore an increased protein surface-solvent interaction in cold-adapted proteins can be hypothesized, at least on the basis of the net serine signal. However, for a confirmation, further sequence analyses are required to gain structural discrimination of solvent exposure. A diverse pattern is detectable for threonine, accounting to a small net gain similar to serine: the surplus of 82 exchanges over alanine significantly increases the polarity, possibly contributing to less hydrophobic interactions within and improved solvent interactions at the surface of proteins. Similarly, the net gain of threonine over asparagine contributes to the overall reduction of the latter and may support better solvent interaction through increased polarity. A surplus of 30 serine exchanges for threonine was detected. At preserved polarity this exchange is likely to increase the accessibility of the hydroxyl group. In contrast, methionine is preferred over threonine (46 M over T), decreasing the polarity. In summary, the multitude and diversity of exchanges within the polar category of amino acids together with the strong signal found in an earlier study [43] point to their importance for thermal adaptation of proteins.

Within the group of unpolar residues no prominent net gains or losses were detected (Figure 5). It should be noted that a moderate gain in the usage of proline for *P. brachycephalum* is detectable, contrasting conclusions of existing views [11,42] as this amino acid causes a large negative impact on structural flexibility. Several amino acid exchanges with conserved functions became apparent in the hydrophobic domain: net gains of 31 alanine in exchange for leucine reflect the preference of shorter residues, i.e., reducing entropic and enthalpic net contributions of side chains to structural stability of protein cores [8,10,11]. The finding of 51 valine over alanine and 65 isoleucine over valine contrast with the global pattern of reduced hydrophobic interaction. However, it is likely that an enlargement in unpolar residue length may enhance the protein–lipid interaction, e.g., within membranes, and must not be in contradiction with the flexibility hypothesis.

As only minor changes in sequence seem to be necessary to adapt kinetic properties, adaptive changes may be difficult to be identified unless proteins of closely related species from different thermal habitats are compared [5,8]. Furthermore, studies based on structural attribution are restricted to proteins with available 3D structures, which is possibly biased towards soluble proteins with enzymatic function as these proteins are overrepresented in common databases. The approach of our study considers all types of expressed proteins within the transcriptome including membrane proteins, structural proteins, etc. without further assumptions.

We claim that our study is less distorted by a probable bias from a specific selection of species, in that we restrict our analyses on model orthologous sequences from two comparable transcriptomes. Furthermore, we focussed on aligned segments, excluding insertions, i.e., we hypothesize that adaptation can be analysed on a purely local basis at single amino acid positions.

In summary, the observed amino acid substitutions represent a global net pattern of molecular shifts in the local, i.e., position-specific amino acid composition for two species of the same family, inhabiting thermally very distinct sites.

### Codon usage

A holistic approach for studying temperature-dependent evolutionary profiles on genetic up to proteomic levels was initially subjected to archaea, bacteria and only some eukaryotes on a large thermal scale [14,44], uncovering trends of a higher GC usage for species with a higher OGT. As the GC content reflects the degree of hydrogen bonding in nucleic acid chain molecules, higher GC contents lead to an increased thermal stability in (deoxy-) ribonuclein acid chains [45,46]. Thermal adaptation was analyzed in structural ribosomal RNAs for prokaryotic *16SrRNA* finding rising GC contents in species with higher OGTs [47]. In vertebrates similar observations were detected in the *18SrRNA*[48] with a trend for higher GC content in endotherm animals compared to poikilotherms showing a correlation to the environmental habitat temperature of the species under study. Similarly, the coding parts of genomes of cold blooded vertebrates and mammals are proposed to be separated by a “major compositional transition” in the GC content, resulting in nearly 100% GC3 levels in mammals [15]. In contrast, genomes of poikilotherm vertebrates are characterized throughout by lower GC3 levels.

Based on the identified segment pairs on the coding sequence level, we focused on a subset of transcript sequences of equal size in both libraries to uncover temperature-related patterns in the codon usage. This filter is supposed to allow for a more specific statistical comparison in that it is not distorted by effects of insertions. We furthermore filtered for highly similar and aligned sequences (80% minimum translated sequence identity) to reduce false pairing. Admittedly, total codon frequencies are on comparable levels for the two species (Figure 7). However, by aggregating all pairwise comparisons in a single Fisher’s test we were able to detect a significant signal (p = 5*10^-4^) of increased AT3 for the Antarctic eelpout (Figure 7). A set of biochemically meaningful hypotheses may be proposed to explain the latter findings. Firstly, under energetically constrained conditions such as in polar environments, proof reading of DNA might be too cost-inefficient and an increment of A/T in the last codon position encoding for synonymous amino acids might be advantageous without having any impact on protein function. A subsequently lowered GC content is therefore discussed for the cold adapted diatom *Fragilariopsis cylindrus* (Thomas Mock, personal communication). Secondly, translation itself can be repressed kinetically through increased GC in codons, over-stabilizing enzymatic transition states within the chain of reaction steps of translation in the cold. Thirdly, to avoid over-stabilized secondary structure elements in messenger RNA in the cold lowering GC content could be an evolutionary resort. For example, the molecular function of cold shock proteins (CSPs) has been described to prevent mRNA from frozen, i.e., over-stabilized hairpin conformations in the cold [49]. An adaptation to the cold by means of reducing GC in the mRNA would imply a reduced pressure to express CSPs permanently at high levels, with implications for metabolic cost.

## Conclusion

So far, many studies have analyzed subsets of orthologous genomic sequences from phylogenetically diverse species to study principles of thermal adaptation on the sequence level. As adjustments in kinetic and thermal properties of enzymes only need subtle changes in primary structure, high genetic identity is pivotal to uncover possible common principles. The present approach includes a comprehensive set of transcriptomic sequence pairs from confamiliar species inhabiting thermally distinct realms differing by about 10°C OGT. Furthermore, the filtering and alignment procedures allow for local analyses on the biomolecular level.

The analysis of synonymous codons uncovers a significant pattern of a higher A/T content for the Antarctic eelpout *P. brachycephalum* compared to the North Sea species *Z. viviparus.* This finding supports the view that cold-adaptation at the DNA/mRNA level took place even on a small thermal gradient in line with results from large scale approaches of distantly related psycrophilic up to hyperthermophilic species [13]. The question arose whether general patterns of cold-adaptation can be identified on the primary structure of proteins. The observed dominating position-specific differences in the primary structure of proteins are pointing to differences in the secondary structure and dynamics, affecting stability and kinetics. Our findings may support the flexibility hypothesis of cold-adaptation, but differences to the existing literature became visible. The former studies revealed some trade-offs for optimization of cold-adapted enzymes like less salt-bridges and more charged side chains for improved solvent interactions. Consequently, cold-adaptation of single proteins uses only a subset of possible alterations. For an understanding of general evolutionary trends in primary protein structure, we provide a more global approach resolving differences between species with similar lifestyles but different thermal adaptation. Protein flexibility at different environmental temperatures may be one adaptation goal whereas for the whole transcriptome other requirements may prevail. The present study should motivate subsequent research with suitable species pairs/groups and full genome coding sequence data to uncover further details of sequence adaptation to the cold.

## Methods

### Phylogenetic analyses of fish

Multiple alignments and phylogenetic trees were generated with MacVector software (Version 10.0.2, MacVector Inc.). For tree construction the neighbour joining method was used, because it avoids assumptions of constant divergence rates among sequences. Node distances were calculated using the Tamura–Nei model, assuming that nucleotide substitutions in different pathways occur with independent probabilities. The sequence data of the genes *COI* and *16SrRNA* were retrieved using the taxonomy browser of the NCBI webpage (http://www.ncbi.nlm.nih.gov/). Detailed information of the used sequences are summarized in Table 3. 

**Table 3:** Summary of sequence data for pyhylogenetic trees

**Species**	**Accession numbers *COI***	**Intercept/ fragment size**	**Accession numbers *16SrRNA***	**Intercept/ fragment size**
*Cyprinus carpio*	*NC_001606*	6,399:7,949	*NC_001606*	2,021:3,701
*Danio rerio*	*NC_002333*	6,425:7,975	*AF036006*	1623
*Gadus morhua*	*GU324197*	652	*AM489716*	1,093:2,757
*Gasterosteus aculeatus*	*EU524639*	652	*NC_003174*	1,087:2,776
*Micromesistius poutassou*	*HQ882656*	579	*NC_015102*	1,090:2,753
*Notothenia coriiceps*	*EU326390*	652	*NC_015653*	16,581:18,273
*Oncorhynchus mykiss*	*GU324178*	652	*NC_001717*	2,088:3,767
*Oryzias latipes*	*AP004421*	5,455:7,011	*AP004421*	1,084:2,757
*Pachycara brachycephalum*	*HQ713113*	652	*Z32732*	542
*Salmo salar*	*GU324184*	652	*NC_001960*	2,093:3,770
*Squalus acanthias*	*EU074608*	652	*NC_002012*	1,093:2,768
*Takifugu rubripes*	*HM102315*	639	*NC_004299*	1,090:2,755
*Tetraodon nigroviridis*	*AP006046*	5,457:7,016	*NC_007176*	1,088:2,764
*Zoarces viviparus*	*EF208064*	1,102	*FJ798757*	588

List of species used for generating the phylogenetic trees in Figure 1 with the mitochondrial sequences for the *COI* gene and structural *16SrRNA* with the respective accession numbers. For some species both sections were extracted from the whole mitochondrial genome due to the availability of the sequence information on the NCBI browser.

### Animal collection and sample preparation of Pachycara brachycephalum

Specimens of *P. brachycephalum* were caught with baited traps at the position 62°10.9' S 58°20.8' W during expedition ANTXV/3 with the RV “Polarstern” in 1998. The animals were brought to the Alfred Wegener Institute in Bremerhaven and kept at 0°C in re-circulated seawater at 34 PSU (practical salinity units). The fish were fed *ad libitum* with *Crangon crangon* once a week*;* feeding was terminated exactly one week before sampling.

For sampling, the fish were anaesthetized by exposure to MS222 (0.2 mg*l^-1^) before being killed. The fish (n=9) had a mean body length of 26 ± 0.83 cm (± SEM) and a mean body weight of 69.77 ± 5.78 g. Tissue samples were quickly excised, frozen instantaneously in liquid nitrogen and stored at −80°C until further processing.

Handling and killing of the fish were conducted in line with the recommendations of the American Veterinary Medical Association (AVMA). The work was approved by a competent German authority (Freie Hansestadt Bremen, reference number 522-27-11/02-00(93).

### cDNA synthesis, normalization and pyrosequencing of Pachycara brachycephalum

Total RNA was extracted from 20–40 mg tissue with the Qiagen RNeasy kit with a modified protocol (proteinase K digestion after homogenisation) according to the manufacturer′s instruction (Qiagen, Hilden, Germany). Quantity and purity of the RNA were determined using the NanoDrop ND 1000 (Peqlab Biotechnologie, Erlangen, Germany). Integrity of the RNA was analysed by capillary electrophoresis (bioanalyser: Agilent, Waldbronn, Germany). Liver- and heart RNA samples of fish were pooled in equal amounts and used for the synthesis of cDNA.

Preparation of a random-primed and normalized cDNA for pyrosequencing was done with 100 μg of pooled total RNA from liver and heart (Vertis, Freising, Germany). Poly(A)+ RNA was prepared and first strand cDNA was amplified with a N6 randomized Primer. 454 sequencing adapter A (5^′^-GCCTCCCTCGCGCCATCAG-3^′^) and B (5^′^- CTGAGCGGGCTGGCAAGGC-3^′^) were ligated to the 5' and 3' ends of the cDNA, followed by an amplification for 21 cycles with a proof-reading enzyme. Normalization was carried out by one cycle of denaturation and reassociation of the cDNA, resulting in N1-cDNA. Reassociated ds-cDNA was subtracted from the remaining ss-cDNA by separation with a hydroxylapatite column. The purified (and normalized) ss-cDNA was amplified with 8 PCR cycles.

The resulting cDNA product was loaded to a preparative agarose gel (1.5%) and fragments in a size range of 450 – 650 bp were eluted for sequencing. The application was performed at Eurofins-MWG, Germany according to [50] using the GS FLX Titanium-technique (MWG-Biotech AG, Ebersberg, Germany). High-density pico reactions were performed on a half of a sequencing run.

### Quality assessment and assembly of cDNA of P. brachycephalum

The sequencing of the normalized cDNA library of *Pachycara brachycephalum* resulted in 481,802 reads with an average read length of 321 bases (Table 1). The reads were preprocessed first with a base calling quality control, followed by a polyA-clipping and a screening for remaining sequencing primer adapters in the 5′as well as in the 3′ends that were truncated. The assembly was done with standard settings for stringency and homology by the Mira Assembler Version 2.9.43 (MWG-Biotech AG, Ebersberg, Germany) according to the instructions by MIRA [51,52]. The high quality reads (338,993) were assembled into 65,565 contigs while 123,038 singlets could not be matched against any other reads. The contigs have a mean length of 487 bases and 22,651 sequences were larger than 500 bases (cf. Figure 2A). The larger contig length compared to *Zoarces viviparus* may be due to different sequencing protocol as we also used the FLX sequencer with a protocol including Titanium chemistry.

### Functional annotation and comparisons of zoarcid cDNA libraries

The assembled contigs of *Pachycara brachycephalum* were loaded into the free software tool BLAST2GO [26] and BLASTed against the NCBI non-redundant database (BLASTx) [53] with an e-value cut-off of 1.0^-3^ and a HSP cut-off length of 33 bases. In total 47,584 Sequences had a BLAST hit, while the e-value distribution (Figure 3A) and the HSP/hit distribution (Figure 3B) reflects the good quality of the *P. brachycephalum* library. We processed the library of contigs of *Zoarces viviparus* in the same way [23] and found in the 53,459 sequences 35,133 with a BLAST result. After the GO-mapping step in BLAST2GO we processed both libraries for annotation with no cut-off for the HSP/hit -coverage, resulting in 19,460 sequences for *P. brachycephalum* and 16,315 sequences for *Z. viviparus.*

The latter sets of sequences were used for evaluating amino acid usage differences and for synonymous/non-synonymous codon usage comparisons. For this, we generated partial sequence translations by rpstBLASTn against a collection of fish-specific orthologies (protein sequences from fiNOG from eggNOG version 3) [54] with e-value below 10^-9^, which yields 52,729 (*P. brachycephalum* and 34,733 (*Z. viviparus*) hits in a total set of 7,374 fiNOG-orthologies. Pairwise alignments between sequences of the two libraries in translated form were then obtained from BLASTP of the obtained fiNOG-compatible translations. 5,352 pairwise alignments (best filtered HSP per fiNOG) with percent-identity above 80% and e-value below 10^-9^ were obtained. Coding sequences for the aligned translated segments were aligned accordingly to the protein alignments. Removal of sequence pairs containing stop-codons and/or ambiguity nucleotide-codes yielded a set of 4,155 segments for further analyses. GC3 comparisons are based on aligned synonymous codon positions with unchanged nucleotides in the first and in the second codon positions.

Amino acid usage has been analysed from these alignments as well as synonymous codon usage (Within Canonical Analysis), both in R [55] with the packages seqinr and ade4 (after [31]). The re-aligned sequence segments contain pairwise information that can be compared in a position-specific way, allowing to quantify preferred amino acid usage, e.g., by counting amino acid specific replacement frequencies for amino acid *i* in *P. brachycephalum* to amino acid *j* in *Z. viviparus* at the same position in the alignment. A non-synonymous replacement imbalance is computed from the matrix.

For a functional characterisation of the libraries, rpstBLASTn was used against the database of metazoan orthologies computed from eggNOG-alignments (version 2, [29]). For comparison with a reference genome of a related fish, we analysed the coding sequences from the genome of *G. aculeatus* (BROADS1, ENSEMBLE Version 1.63 at EMBL) in the same way. Best hits to meNOG orthologies with e-value below 10^-20^ were kept, pooling functional annotations into COG/KOG categories.

### Access to data

The transcriptome of *P. brachycephalum* is published in the sequencing-read archive (SRA) at NCBI under Accession SRA049761.

## Competing interests

The authors declare that they have no competing interests.

## Authors’ contributions

HSW participated in the concept and experimental design, carried out the preparation of samples, the annotation of contigs, participated in the interpretation of data and drafted the manuscript. ML conceived the concept and designed the experiment, participated in the interpretation of data and helped to draft the manuscript. SF contributed the idea of sequence-based signatures to this study, developed the concept and implementation of the sequence analyses and helped to draft the manuscript. All authors read and approved the final manuscript.

## References

[B1] Pörtner HO Oxygen- and capacity-limitation of thermal tolerance: a matrix for integrating climate-related stressor effects in marine ecosystems J Exp Biol 2010 213 6 881 893 10.1242/jeb.037523 20190113

[B2] Pörtner HO Peck L Somero G Thermal limits and adaptation in marine Antarctic ectotherms: an integrative view Philos Trans R Soc B-Biol Sci 2007 362 1488 2233 2258 10.1098/rstb.2006.1947 PMC2443174 17553776

[B3] Hofmann GE Lund SG Place SP Whitmer AC Some like it hot, some like it cold: the heat shock response is found in New Zealand but not Antarctic notothenioid fishes J Exp Mar Biol Ecol 2005 316 1 79 89 10.1016/j.jembe.2004.10.007

[B4] Bilyk K DeVries A Freezing avoidance of the Antarctic icefishes (Channichthyidae) across thermal gradients in the Southern Ocean Polar Biol 2010 33 2 203 213 10.1007/s00300-009-0697-z

[B5] Somero GN Temperature Relationships: From Molecules to Biogeography Comprehensive Physiology 2011, Supplement 30: Handbook of Physiology, Comparative Physiology 1997 1391 1444 First published in print 1997

[B6] Bentahir M Feller G Aittaleb M Lamotte-Brasseur J Himri T Chessa JP Gerday C Structural, kinetic, and calorimetric characterization of the cold-active phosphoglycerate kinase from the antarctic Pseudomonas sp. TACII18 J Biol Chem 2000 275 15 11147 11153 10.1074/jbc.275.15.11147 10753921

[B7] Lonhienne T Zoidakis J Vorgias CE Feller G Gerday C Bouriotis V Modular structure, local flexibility and cold-activity of a novel chitobiase from a psychrophilic Antarctic bacterium J Mol Biol 2001 310 2 291 297 10.1006/jmbi.2001.4774 11428890

[B8] Somero GN Adaptation of enzymes to temperature: searching for basic "strategies" Comp Biochem Physiol B Biochem Mol Biol 2004 139 3 321 333 10.1016/j.cbpc.2004.05.003 15544958

[B9] Somero GN The physiology of climate change: how potentials for acclimatization and genetic adaptation will determine 'winners' and 'losers' J Exp Biol 2009 213 6 912 920 20190116 10.1242/jeb.037473

[B10] Marx JC Blaise V Collins T Damico S Delille D Gratia E Hoyoux A Huston AL Sonan G Feller G A perspective on cold enzymes: Current knowledge and frequently asked questions Cell Mol Biol 2004 50 5 643 655 15559980

[B11] Feller G Gerday C Psychrophilic enzymes: hot topics in cold adaptation Nat Rev Micro 2003 1 3 200 208 10.1038/nrmicro773 15035024

[B12] Georlette D Blaise V Collins T D'Amico S Gratia E Hoyoux A Marx JC Sonan G Feller G Gerday C Some like it cold: biocatalysis at low temperatures FEMS Microbiol Rev 2004 28 1 25 42 10.1016/j.femsre.2003.07.003 14975528

[B13] Tekaia F Yeramian E Evolution of proteomes: fundamental signatures and global trends in amino acid compositions BMC Genomics 2006 7 1 307 10.1186/1471-2164-7-307 17147802 PMC1764020

[B14] Tekaia F Yeramian E Dujon B Amino acid composition of genomes, lifestyles of organisms, and evolutionary trends: a global picture with correspondence analysis Gene 2002 297 1–2 51 60 12384285 10.1016/s0378-1119(02)00871-5

[B15] Bernardi G The compositional evolution of vertebrate genomes Gene 2000 259 1–2 31 43 11163959 10.1016/s0378-1119(00)00441-8

[B16] Cruveiller S Jabbari K D'Onofrio G Bernardi G Different hydrophobicities of orthologous proteins from Xenopus and human Gene 1999 238 1 15 21 10.1016/S0378-1119(99)00259-0 10570979

[B17] Bernardi G Hughes S Mouchiroud D The major compositional transitions in the vertebrate genome J Mol Evol 1997 44 S44 S51 10.1007/PL00000051 9071011

[B18] Salem M Rexroad C Wang J Thorgaard G Yao J Characterization of the rainbow trout transcriptome using Sanger and 454-pyrosequencing approaches BMC Genomics 2010 11 1 564 10.1186/1471-2164-11-564 20942956 PMC3091713

[B19] Johansen SD Karlsen BO Furmanek T Andreassen M Jørgensen TE Bizuayehu TT Breines R Emblem Å Kettunen P Luukko K RNA deep sequencing of the Atlantic cod transcriptome Comp Biochem Physiol D Genom Proteom 2011 6 1 18 22 10.1016/j.cbd.2010.04.005 20493789

[B20] Anderson ME Fedorov VV Family Zoarcidae Swainson 1839 - eelpouts California Academy of Sciences - Annotated Checklists of Fishes 2004 34 1 58

[B21] Zakhartsev MV De Wachter B Sartoris FJ Pörtner HO Blust R Thermal physiology of the common eelpout (*Zoarces viviparus*) J Comp Physiol B 2003 173 5 365 378 10.1007/s00360-003-0342-z 12774171

[B22] Pörtner HO Knust R Climate Change Affects Marine Fishes Through the Oxygen Limitation of Thermal Tolerance Science 2007 315 5808 95 97 10.1126/science.1135471 17204649

[B23] Kristiansson E Asker N Forlin L Larsson DJ Characterization of the *Zoarces viviparus* liver transcriptome using massively parallel pyrosequencing BMC Genomics 2009 10 1 345 10.1186/1471-2164-10-345 19646242 PMC2725146

[B24] van Dijk PL Tesch C Hardewig II Pörtner HO Physiological disturbances at critically high temperatures: a comparison between stenothermal antarctic and eurythermal temperate eelpouts (Zoarcidae) J Exp Biol 1999 202 Pt 24 3611 3621 10574738 10.1242/jeb.202.24.3611

[B25] Brodte E Knust R Pörtner HO Temperature-dependent energy allocation to growth in Antarctic and boreal eelpout (Zoarcidae) Polar Biol 2006 30 1 95 107 10.1007/s00300-006-0165-y

[B26] Conesa A Goetz S Garcia-Gomez JM Terol J Talon M Robles M Blast2GO: a universal tool for annotation, visualization and analysis in functional genomics research Bioinformatics 2005 21 18 3674 3676 10.1093/bioinformatics/bti610 16081474

[B27] Altschul SF Gish W Miller W Myers EW Lipman DJ Basic local alignment search tool J Mol Biol 1990 215 3 403 410 2231712 10.1016/S0022-2836(05)80360-2

[B28] Trachana K Larsson TA Powell S Chen W-H Doerks T Muller J Bork P Orthology prediction methods: A quality assessment using curated protein families Bioessays 2011 33 10 769 780 10.1002/bies.201100062 21853451 PMC3193375

[B29] Muller J Szklarczyk D Julien P Letunic I Roth A Kuhn M Powell S von Mering C Doerks T Jensen LJ eggNOG v2.0: extending the evolutionary genealogy of genes with enhanced non-supervised orthologous groups, species and functional annotations Nucl Acids Res 2010 38 suppl 1 D190 D195 19900971 10.1093/nar/gkp951 PMC2808932

[B30] Wang G-Z Lercher M Amino acid composition in endothermic vertebrates is biased in the same direction as in thermophilic prokaryotes BMC Evol Biol 2010 10 1 263 10.1186/1471-2148-10-263 20807394 PMC2939578

[B31] Charif D Thioulouse J Lobry JR Perrière G Online synonymous codon usage analyses with the ade4 and seqinR packages Bioinformatics 2005 21 4 545 547 10.1093/bioinformatics/bti037 15374859

[B32] Garland TJ Adolph SC Why Not to Do Two-Species Comparative Studies: Limitations on Inferring Adaptation Physiol Zool 1994 67 4 797 828

[B33] Ikemura T Codon usage and tRNA content in unicellular and multicellular organisms Mol Biol Evol 1985 2 1 13 34 3916708 10.1093/oxfordjournals.molbev.a040335

[B34] Ozato K Shin D-M Chang T-H Morse HC TRIM family proteins and their emerging roles in innate immunity Nat Rev Immunol 2008 8 11 849 860 10.1038/nri2413 18836477 PMC3433745

[B35] Tort LI Balasch JC Mackenzie S Fish immune system. A crossroads between innate and adaptive responses Immunología 2003 22 3 277 287

[B36] Star B Nederbragt AJ Jentoft S Grimholt U Malmstrom M Gregers TF Rounge TB Paulsen J Solbakken MH Sharma A The genome sequence of Atlantic cod reveals a unique immune system Nature 2011 477 7363 207 210 10.1038/nature10342 21832995 PMC3537168

[B37] Brodte E Graeve M Jacob U Knust R Pörtner HO Temperature-dependent lipid levels and components in polar and temperate eelpout (Zoarcidae) Fish Physiol Biochem 2008 34 3 261 274 10.1007/s10695-007-9185-y 18665464

[B38] Lund ED Sidell BD Neutral lipid compositions of antarctic fish tissues may reflect use of fatty acyl substrates by catabolic systems Mar Biol 1992 112 3 377 382 10.1007/BF00356282

[B39] Windisch HS Kathöver R Pörtner H-O Frickenhaus S Lucassen M Thermal acclimation in Antarctic fish: Transcriptomic profiling of metabolic pathways Am J Physiol Regul Integr Comp Physiol 2011 301 5 R1453 R1466 10.1152/ajpregu.00158.2011 21865546

[B40] Lobry JR Necsulea A Synonymous codon usage and its potential link with optimal growth temperature in prokaryotes Gene 2006 385 128 136 16989961 10.1016/j.gene.2006.05.033

[B41] Gu J Hilser VJ Sequence-Based Analysis of Protein Energy Landscapes Reveals Nonuniform Thermal Adaptation within the Proteome Mol Biol Evol 2009 26 10 2217 2227 10.1093/molbev/msp140 19592668 PMC2912464

[B42] Feller G Gerday C Psychrophilic enzymes: molecular basis of cold adaptation Cell Mol Life Sci 1997 53 10 830 841 10.1007/s000180050103 9413552 PMC11147173

[B43] Methé BA Nelson KE Deming JW Momen B Melamud E Zhang X Moult J Madupu R Nelson WC Dodson RJ The psychrophilic lifestyle as revealed by the genome sequence of Colwellia psychrerythraea 34H through genomic and proteomic analyses Proc Natl Acad Sci USA 2005 102 31 10913 10918 10.1073/pnas.0504766102 16043709 PMC1180510

[B44] Hickey D Singer G Genomic and proteomic adaptations to growth at high temperature Genome Biol 2004 5 10 117 10.1186/gb-2004-5-10-117 15461805 PMC545586

[B45] Wada A Suyama A Local stability of DNA and RNA secondary structure and its relation to biological functions Prog Biophys Mol Biol 1986 47 2 113 157 10.1016/0079-6107(86)90012-X 2424044

[B46] Russell AP Holleman DS The thermal denaturatlion of DNA: average length and composition of denatured areas Nucl Acids Res 1974 1 8 959 978 10.1093/nar/1.8.959 10793728 PMC343404

[B47] Wang H-C Xia X Hickey D Thermal Adaptation of the Small Subunit Ribosomal RNA Gene: A Comparative Study J Mol Evol 2006 63 1 120 126 10.1007/s00239-005-0255-4 16786438

[B48] Varriale A Torelli G Bernardi G Compositional properties and thermal adaptation of 18S rRNA in vertebrates RNA 2008 14 8 1492 1500 10.1261/rna.957108 18567811 PMC2491464

[B49] Schindelin H Marahiel MA Heinemann U Universal nucleic acid-binding domain revealed by crystal structure of the B. subtilis major cold-shock protein Nature 1993 364 6433 164 168 10.1038/364164a0 8321288

[B50] Margulies M Egholm M Altman WE Attiya S Bader JS Bemben LA Berka J Braverman MS Chen YJ Chen Z Genome sequencing in microfabricated high-density picolitre reactors Nature 2005 437 7057 376 380 16056220 10.1038/nature03959 PMC1464427

[B51] Chevreux B Wetter T Suhai S Genome Sequence Assembly Using Trace Signals and Additional Sequence Information. In: Computer Science and Biology Proceedings of the German Conference on Bioinformatics GCB 99 Hannover, Germany: Citeseer 45 56

[B52] Chevreux B Pfisterer T Drescher B Driesel AJ Müller WEG Wetter T Suhai S Using the miraEST Assembler for Reliable and Automated mRNA Transcript Assembly and SNP Detection in Sequenced ESTs Genome Res 2004 14 6 1147 1159 10.1101/gr.1917404 15140833 PMC419793

[B53] Altschul SF Madden TL Schaffer AA Zhang J Zhang Z Miller W Lipman DJ Gapped BLAST and PSI-BLAST: a new generation of protein database search programs Nucleic Acids Res 1997 25 17 3389 3402 10.1093/nar/25.17.3389 9254694 PMC146917

[B54] Powell S Szklarczyk D Trachana K Roth A Kuhn M Muller J Arnold R Rattei T Letunic I Doerks T eggNOG v3.0: orthologous groups covering 1133 organisms at 41 different taxonomic ranges Nucl Acids Res 2011 40 D1 D284 D289 22096231 10.1093/nar/gkr1060 PMC3245133

[B55] R-Development-Core-Team R: A language and environment for statistical computing 2011 Vienna R Foundation for Statistical Computing, ISBN 3-900051-07-0, http://www.R-project.org

